# Human Brain In Vitro Model for Pathogen Infection-Related Neurodegeneration Study

**DOI:** 10.3390/ijms25126522

**Published:** 2024-06-13

**Authors:** Yuwei Yan, Ann-Na Cho

**Affiliations:** 1 School of Biomedical Engineering, Faculty of Engineering, The University of Sydney, Darlington, NSW 2008, Australia; yuwei.yan@sydney.edu.au; 2 The University of Sydney Nano Institute (Sydney Nano), The University of Sydney, Camperdown, NSW 2050, Australia; 3 Charles Perkins Centre, The University of Sydney, Camperdown, NSW 2006, Australia

**Keywords:** pathogen–host interaction, infection models, in vitro human brain models, brain organoids, organ-on-chip, human brain population

## Abstract

Recent advancements in stem cell biology and tissue engineering have revolutionized the field of neurodegeneration research by enabling the development of sophisticated in vitro human brain models. These models, including 2D monolayer cultures, 3D organoids, organ-on-chips, and bioengineered 3D tissue models, aim to recapitulate the cellular diversity, structural organization, and functional properties of the native human brain. This review highlights how these in vitro brain models have been used to investigate the effects of various pathogens, including viruses, bacteria, fungi, and parasites infection, particularly in the human brain cand their subsequent impacts on neurodegenerative diseases. Traditional studies have demonstrated the susceptibility of different 2D brain cell types to infection, elucidated the mechanisms underlying pathogen-induced neuroinflammation, and identified potential therapeutic targets. Therefore, current methodological improvement brought the technology of 3D models to overcome the challenges of 2D cells, such as the limited cellular diversity, incomplete microenvironment, and lack of morphological structures by highlighting the need for further technological advancements. This review underscored the significance of in vitro human brain cell from 2D monolayer to bioengineered 3D tissue model for elucidating the intricate dynamics for pathogen infection modeling. These in vitro human brain cell enabled researchers to unravel human specific mechanisms underlying various pathogen infections such as SARS-CoV-2 to alter blood-brain-barrier function and *Toxoplasma gondii* impacting neural cell morphology and its function. Ultimately, these in vitro human brain models hold promise as personalized platforms for development of drug compound, gene therapy, and vaccine. Overall, we discussed the recent progress in in vitro human brain models, their applications in studying pathogen infection-related neurodegeneration, and future directions.

## 1. Introduction

The brain is the most complex organ in the body, consisting of a diverse cell population and specific microenvironments (e.g., extracellular matrix, fluids), which are managed by independent and hierarchical interconnections of multiple regions in the central nervous system (CNS) [1,2,3]. The brain microenvironment is a complex and dynamic entity, crucial for both normal brain function and the development of various pathologies [4,5,6]. During the last few years of the pandemic, there has been a lot of emphasis on pathology studies and their linkage to neurological symptoms [7]. Pathology provides crucial insights into the fundamental and functional alterations in the neural systems, under various pathological conditions in the brain [7]. Over the past decades, in vitro brain models have shown promise as humanized disease model platforms by fully leveraging their similarity to in vivo human brain physiological and pathological conditions [8]. Moreover, these in vitro human brain models suggest minimal ethical issues associated with extensive animal model usage and post-mortem tissues [8,9,10]. From 2D monolayer cell cultures to advanced 3D models including brain organoids, organ-on-chip systems, and bioengineered tissue models, in vitro human models mimicking human brain tissues have shown great achievements in neuroscience, especially in pathology through continuous technological developments. Those human models facilitate a deeper understanding of pathogen infection mechanisms, the discovery of diagnostic markers, and the identification of potential therapeutic targets for neurological symptoms [10]. In this review, we will introduce the cellular population comprising the human brain, various in vitro brain models to model those populations and the native brain microenvironment, and their applications in pathogen infection studies focusing on pathogen–host interactions and subsequent neurodegeneration and neurological symptoms.

## 2. Cellular Population of Central Nervous System

**Figure 1:**
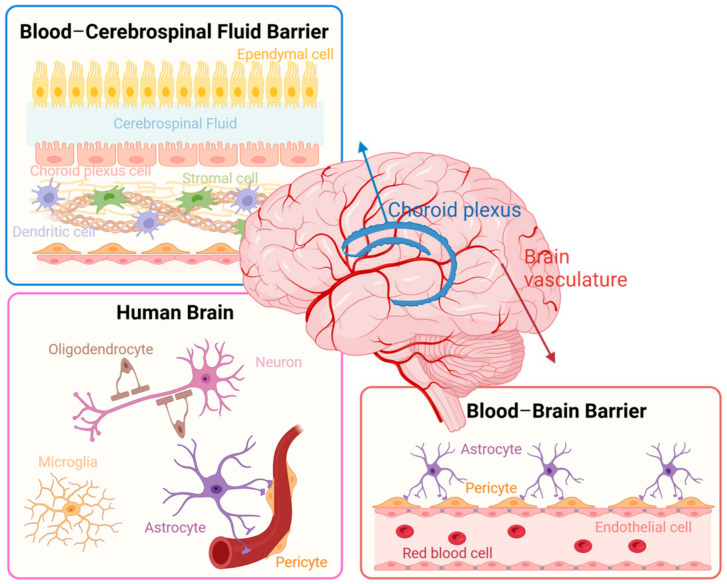
Diverse cellular population of central nervous system (CNS). Brain cells include neurons, astrocytes, oligodendrocytes, microglia, and pericytes. Blood–brain barrier (BBB) is formed by brain microvascular endothelial cells (BMECs) forming tight junctions with astrocytes, and pericytes. Blood–cerebrospinal fluid (CSF) barrier contains ependymal cells, choroid plexus cells, stromal cells, and dendritic cells.

The human brain consists of approximately 100 billion neurons, including 3313 subcluster neurons, and approximately 1 trillion glial cells (Figure 1) [11,12,13,14]. Neurons are considered the key players in brain function, using electrical and chemical signal transmission between neurons, and other cells in the CNS and the entire body [11]. Glial cells provide support to neurons, including astrocytes, which promote neuronal survival and maintain brain homeostasis; microglia, which exhibit immune system-like activity and protect the brain from infections; oligodendrocytes, involved in neuronal myelination; and ependymal cells, which produce cerebrospinal fluid (Figure 1).

### 2.1. Subtype Neurons

Neurons are the primary cells responsible for transmitting electrical signals in the brain. The communication and signal transmission of neurons is medicated by synapses, specialized junctions between two neurons, where chemical and electrical signals are transmitted [15]. Based on their effect on the brain, neurons can be broadly classified as excitatory neurons, which can stimulate neuronal activities, and inhibitory neurons which suppress activities [16]. Excitatory neurons transmit signals that activate other neurons through excitatory neurotransmitters (e.g., glutamate), while inhibitory neurons transmit signals that inhibit the activity of other neurons by releasing inhibitory neurotransmitters such as gamma-aminobutyric acid (GABA) [17]. Therefore, the balance between excitatory and inhibitory neurons is crucial to maintaining the neural circuit [18].

Excitatory projection neurons are classified based on their efferent targets, with the intratelencephalic (IT) type projecting to other cortical areas and the striatum, while the pyramidal tract (PT) type projects to more distant targets like the thalamus, midbrain, brainstem, and spinal cord [19]. IT neurons facilitate intracortical communication and corticostriatal connections, which are essential for integrating sensory information and coordinating complex behaviors. PT neurons are particularly important for sensory perception and motor execution, encoding separate streams of information necessary for the perception of tactile or visual stimuli and for initiating motor responses [20]. A healthy human brain maintains a constant excitatory/inhibitory (E/I) ratio to sustain normal function and stability [21]. Imbalances in the E/I ratio can lead to neurological disorders such as Autism Spectrum Disorder (ASD) and schizophrenia.

Cortical interneurons play a crucial role in maintaining the balance of E/I within the neural circuits by using GABA as a key transmitter [22]. Various subtypes of inhibitory neurons, including those expressing three different calcium-binding proteins parvalbumin (PV), calbindin (CB), and calretinin (CR) in different populations; somatostatin (SST); and vasoactive intestinal peptide (VIP) are classified with various morphology and electrophysiology [22,23,24,25,26]. PV interneurons are involved in controlling neuron spiking and network oscillations, typically targeting the area around the cell body of pyramidal neurons, while SST interneurons target the distal dendritic regions and are key in regulating local conductivity between the synapse and dendritic spine [27]. PV interneurons are implicated in promoting feedforward inhibition, thereby facilitating low-frequency spike synchronization, while SST interneurons enhance feedback inhibition, crucial for high-frequency spike synchronization. This intricate balance between PV and SST neurons is essential for the nuanced regulation of synaptic feedback [27,28,29,30]. VIP inhibitory neurons exhibit high spine densities on their dendrites and the presence of excitatory synaptic contacts. This dense connectivity and the presence of multiple excitatory synapses on individual spines suggest that VIP interneurons are efficiently depolarized by clustered synaptic inputs, facilitating their role in cortical plasticity [25,31]. Research has shown that alterations in PV-inhibitory neurons are central to cognitive dysfunction in schizophrenia and the density of perineuronal nets (PNNs) regulate PV cell stability and function [32].

### 2.2. Astrocyte

Astrocytes are star-shaped cells with numerous branches essential for providing structural support in the CNS by forming a porous scaffold [33,34]. They maintain the homeostasis of the extracellular matrix, monitoring the CNS microenvironment and regulating potassium ion concentrations [35]. Additionally, astrocytes aid in neurotransmitter clearance at synapse clefts through neurotransmitter transporters, Excitatory Amino Acid Transporters (EAATs) for glutamates, and Gamma-Aminobutyric Acid Transporters (GATs) for GABA, preventing glutamate toxicity by absorbing excess glutamate and supporting neuronal function by releasing glutamate in a controlled manner. This balance is crucial for brain health maintenance; therefore, it could be targeted for treating extensive glutamate levels associated with CNS diseases [36]. Astrocytes also play a significant role in the blood–brain barrier (BBB) as one of the major components [37]. They are central to maintaining brain health; however, in disease conditions, astrocytes can either protect or damage the nervous system. In Alzheimer’s disease (AD), a cycle of amyloid-beta (Aβ) generation between astrocytes and neurons leads to chronic neuroinflammation, and astrocytes can trigger apoptosis in neurons in progressed stages [38]. Astrocytes show a severe decrease in glutamate transporters, linked to neurotoxicity in Amyotrophic Lateral Sclerosis (ALS) and abnormal astrocytic homeostasis can contribute to the development of seizures [39]. Eugenin et al. found that human immunodeficiency virus (HIV)-infected astrocytes can disrupt BBB integrity by spreading toxic signals to neighboring uninfected astrocytes, despite their low infection rate [40]. Bohmwald et al. revealed that astrocytes exhibit an inflammatory response, characterized by the increased production of pro-inflammatory cytokines when infected by the human respiratory syncytial virus (hRSV) [41]. Astrocytes exhibit diverse roles in CNS inflammation, where they can both mitigate and exacerbate neurodegenerative conditions by restricting peripheral immune cell influx and supporting tissue repair through neurotrophic factor productions. Conversely, they can also promote neurodegeneration by recruiting peripheral inflammatory cells (e.g., macrophages) or through their own neurotoxic activities [42].

### 2.3. Oligodendrocyte

An oligodendrocyte is formed with a soma and several branches that wrap around the axon of neurons, insulating neurons to transmit electrical signals rapidly and precisely. Different oligodendrocytes can wrap around axons in multiple segments, forming structures called myelin sheaths [43,44]. Recent research also indicates that oligodendrocytes play vital roles in learning and memory functions [45]. The myelin sheath is not merely an insulating layer but also a metabolically active structure involved in transporting macromolecules. The stability and molecular composition of myelin, with its high lipid content and specific proteins like myelin basic protein (MBP) and proteolipid protein (PLP), contributes to its function and longevity. Additionally, myelin is dynamic and can undergo structural changes in response to environmental stimuli, indicating a form of neural plasticity [46,47]. Moreover, myelin is a key component of white matter and oligodendrocyte progenitor cells contribute to the remyelination and repair of white matter by proliferating and generating new oligodendrocytes [48]. This repair process is essential for the recovery of function and plasticity in neuronal circuits after white matter degeneration including traumatic brain injuries (TBIs) [49]. The hypoxia-inducible factor (HIF) within oligodendrocyte precursor cells (OPCs) regulates the relationship between the formation of myelin and the development of blood vessels after birth. This coordination ensures that the onset of myelination is timed with the establishment of adequate vasculature to support the metabolic needs of myelinating oligodendrocytes and the axons they ensheathe [50]. In white matter abnormalities, damage to myelin and changes in oligodendrocyte function have been observed by extensive DNA repair mechanisms in the pathological condition of AD [51]. Research shows that oligodendrocytes can survive an acute coronavirus infection and continue to induce inflammatory responses within the CNS for an extended period [52]. This finding suggests a potential mechanism by which viral infections could lead to chronic inflammation and possibly to demyelinating diseases such as multiple sclerosis (MS). In the Zika virus infection model, oligodendrocytes are susceptible to Zika virus infection, which can cause cell death and subsequent neurodevelopmental delays in infants who were exposed to the virus in utero but born asymptomatic. The findings underscore the importance of long-term surveillance for children with prenatal Zika virus exposure to monitor and address the potential delayed onset of neurological complications [53].

### 2.4. Microglia

Microglia function as resident immune cells in the CNS. In the resting state, they have a morphology similar to astrocytes, while they change their patterns to resemble macrophages in an active state during inflammatory conditions caused by infection from pathogens or injuries in the CNS [54]. Microglia are derived from the yolk sac and have a dual role in neurodegenerative diseases as both protectors and contributors to pathology [55]. Dysregulation of microglial functions, such as sensing and housekeeping, and can lead to an excessive inflammatory response which eventually causing neuronal damage. Therefore, targeting microglial functions could be a promising strategy to improve brain health in the immunogenicity environment including demyelination conditions [56,57,58]. Under normal conditions, microglial proliferation is slow, whereas their population can increase through both the proliferation of resident microglia and the recruitment of bone marrow-derived immune cells under pathological conditions. Unlike other macrophages that are typically replenished by bone marrow-derived cells, microglia are long-lived, self-renewing, and not replaced by peripheral cells under normal conditions. This self-renewal ability allows them to expand in response to certain environmental cues [59,60]. During Human immunodeficiency virus (HIV) type 1 infection, the viral protein Tat can stimulate microglia release of excessive glutamate, causing excitotoxicity, and also promote microglial migration and phagocytosis, resulting in the loss of neuronal synapses. Another viral protein Gp120 can activate microglia, influencing processes like phagocytosis and cytokine secretion, which are implicated in neuronal damage [61]. Jeong et al. find that microglia can be directly infected by SARS-CoV-2, leading to proinflammatory responses and cell death. The virus triggers an M1-like proinflammatory activation in human microglial cells, which is followed by apoptotic cell death through both intrinsic and extrinsic pathways [62]. Carrillo et al. find that microglia become activated during *Toxoplasma gondii* (*T. gondii*) infection, by ensheathing both excitatory and inhibitory neurons, and suggesting that microglia could contribute to synaptic loss by displacing or phagocytosing synaptic elements [63]. During *Trypanosoma brucei* (*TbR*) infection, microglia undergo significant transcriptional changes, including an upregulation of genes involved in antigen presentation and a response to chemokine signaling, which lead to an inflammatory response and the adoption of an infection-associated phenotype [64]. A unique type of microglia, named disease-associated microglia (DAM), restricts the development of AD by triggering the receptor expressed on myeloid cells 2 inhibitory checkpoint pathways in microglia [65].

### 2.5. Brain Microvascular Endothelial Cells and Stromal Cells

Brain microvascular endothelial cells (BMECs) form the capillaries in the brain, acting as key elements of the BBB, which can prevent toxins and other cells in the bloodstream from entering the brain [66,67]. BMECs are involved in regulating BBB permeability, and their dysfunction can lead to the infiltration of immune cells and plasma proteins into the brain, exacerbating neuroinflammation [68]. In conditions like AD, dysfunction of BMECs leads to BBB breakdown, allowing harmful substances, including amyloid-beta (Aβ), to accumulate in the brain and contribute to the formation of plaques, a characteristic feature of AD [69]. Studies have shown that abnormalities in BMECs, such as changes in tight junction proteins, may be associated with the pathology of schizophrenia [66]. Mitochondrial oxidative stress in BMECs leads to BBB damage by impairing the function of tight junctions and adhesion junctions, which are crucial for maintaining BBB integrity. This stress can induce abnormal angiogenesis and inhibit the endothelial nitric oxide synthase (eNOS) pathway, leading to neurovascular decoupling and further aggravating BBB damage [70]. The development of viral vectors, such as AAV-BR1, that specifically target BMECs, has opened up new possibilities for delivering therapeutic genes directly to the brain [71]. Novel in vitro models of the brain neurovascular unit (NVU) that include BMECs have been developed to study the pathogen and potential therapeutics. Xue et al. developed a triple cell co-culture model of the NVU that closely mimics the in vivo environment, exhibiting enhanced BBB function. The model’s response to anoxia-reoxygenation paralleled the pathological changes observed in cerebral diseases, indicating its potential for studying brain cells and BBB pathology [72]. The microvascular endothelial cell (EC) pairs model has been developed to predict BBB permeability by observing structural changes in human BMECs. High-permeability cell pairs exhibited nuclear elongation, loss of junction proteins, and increased actin stress fiber formation, indicative of increased contractility and barrier leakiness [73].

Stromal cells in the brain are integral to the organ’s structure, providing support and maintaining the specific form of the brain [74]. There are many types of stromal cells, and in the brain, the most widely used are fibroblasts and pericytes, which are essential for maintaining the integrity of the BBB [75,76,77]. Pericytes are embedded within the basement membrane and play a pivotal role in regulating the permeability of the BBB by affecting the structure and function of tight junctions. Furthermore, they are involved in the crosstalk with other cellular components of the BBB, such as astrocytes, to facilitate vascular maturation and angiogenesis [78]. In the absence of pericytes, molecules (e.g., ALCAM) are upregulated which can increase leukocyte migration across ECs, potentially exacerbating conditions like MS [79]. Pericyte deficiency in the brain also results in reduced brain capillary perfusion and cerebral blood flow by changing the diameter of capillaries, which can cause chronic perfusion stress and hypoxia. This process shows that even a modest loss of pericytes can initiate vascular damage in young mice, which becomes more pronounced with age [80]. Nakagawa et al. find that pericytes can be infected by HIV-1, by expressing the necessary receptors that allow HIV-1 to enter and infect, which leads to increased levels of the inflammatory marker IL-6 and disrupts the integrity of the BBB [81]. The infection of SARS-CoV-2 in pericytes is associated with perivascular inflammation and a compromised BBB, as indicated by the leakage of fibrinogen. Consequently, the cerebrospinal fluid of infected individuals shows reduced levels of the pericyte marker, PDGFRβ, suggesting a disturbance in pericyte homeostasis [82]. Pericytes help to control inflammation by reducing the activation of ECs during infections with bacteria like *Brucella ovis*, *Listeria monocytogenes*, and *Citrobacter rodentium*. Increased inflammation and higher bacterial levels in organs have been shown in mouse models without pericytes [83]. Additionally, they have been suggested to possess pluripotent characteristics, with the potential to differentiate into multiple brain cell lineages [84,85,86,87].

## 3. In Vitro Model of Human Brain

**Figure 2:**
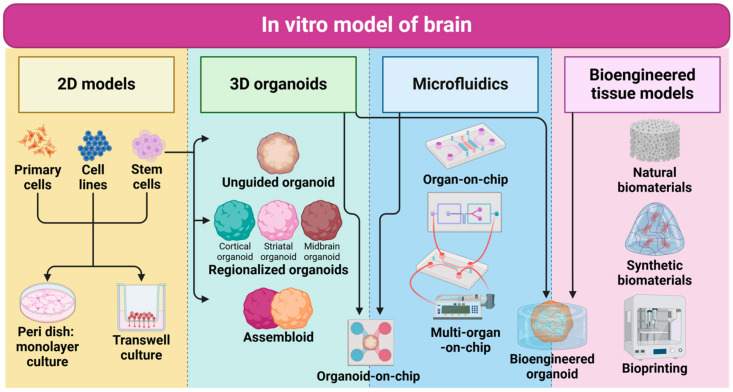
In vitro model of human brain. 2D models include cell sources of primary cells, cell lines, and stem cells; moreover, culture platform includes monolayer culture on petri dish and transwell. 3D organoids are categorized into unguided organoids, regionalized organoids, and assembloids, additionally can also be integrated with microfluidics and/or bioengineered tissue models to generate organoid-on-chip and/or bioengineered organoids. Microfluidic model includes organ-on-chip and multi-organ-on-chip model And bioengineered tissue models cover natural and synthetic biomaterials, and technology of bioprinting.

In past decades, in vitro models to recapitulate the human brain have been extensively developed to study brain development, homeostasis, and disease conditions (e.g., neurodegenerative diseases) [88,89]. These models are categorized into 2D monolayer cultures, 2D transwells, 3D organoids, organ-on-chips, and bioengineered tissue models aiming to resemble the complexity of the native brain including molecular profiles, cellular populations, function, interactions, and structures. A 2D monolayer cell culture and transwell systems are useful for basic kinetic studies for gaining homogenous neuronal responses in drug testing but lack the complexity of the human brain tissue such as cellular diversity, spatial orientation, and 3D morphologies [90,91]. Therefore, a significant advance in cell and tissue engineering technology to generate more complicated human brain model systems including 3D organoids, organ-on-chip, and bioengineered tissue models offer a more accurate representation of the human brain’s unique nature (Table 1). Recent advancements in in vitro human brain models suggest variable choices for multiple studies in neurodevelopment, neurological disorders, and pathogen infection-driven neurodegeneration (Figure 2).

### 3.1. 2D Monolayer Cultures

Primary cells retain the similar properties of native in vivo brain tissues since they are directly derived from tissues [92]. However, the fundamental drawbacks of primary cells lie in their challenging accessibility, as those cells can only be collected from human brain tissue through surgical procedures or postmortem resources [93]. Although these cells carry the same pathological properties and specific pathology of the patient, due to the cell proliferation and passage limit, the resources are difficult to maintain throughout the experiment, and thus inconsistent results will be produced, yielding unstandarized findings [94]. Primary cells also have lower growth rates and limited longevity than in vivo cells, further requiring optimal cultural conditions for each cellular source [95]. The most critical limitation is that cells are collected from only one timeline; thus, there is limited temporal information including lack of biological resources from different time points of pathological progress from early onset to long-term symptoms [94].

Immortalized cell lines can acquire the ability to proliferate indefinitely by the introduction of telomerase reverse transcriptase (TERT) genes [96]; therefore, it produces the cells more robustly and makes it easier to manipulate than primary cells. Different immortal cell lines have been used for in vitro models, including HT-22, which models mouse hippocampal neurons [97], PC-12, commonly used for models of neurotoxicity and neuroinflammation [98], and SH-SY5Y and LUHMES, used for dopaminergic neuronal studies [93,99,100]. However, the drawbacks of using cell lines include their genetic modification, which may lead to altered physiological properties not representative of the in vivo state, further compounded by changes induced through repeated passaging over time [101].

Stem cells are a type of cells with the ability to differentiate into various specialized cell types within the body. Neural stem cells (NSCs) are multipotent stem cells that can differentiate into various cell types found in the CNS, including neurons, astrocytes, and oligodendrocytes [102]. These cells are found in specific areas of the brain known as germinal niches, such as the subventricular zone (SVZ) of the lateral ventricles (LVs) and the subgranular zone (SGZ) of the hippocampal dentate gyrus, which support neurogenesis and gliogenesis throughout adult life [103]. Ependymal cells, lining the ventricles of the brain, have been identified as neural stem cells. In response to spinal cord injury, ependymal cells proliferate to produce migrating cells that differentiate into astrocytes, which are involved in scar formation [104]. The fate choice of NSCs, whether to become neurons or glial cells, is influenced by niche signals, with astrocyte-derived WNT promoting neuronal fate and bone morphogenic proteins (BMPs) favoring glial differentiation [105].

Human pluripotent stem cells (hPSCs) can be classified into two types, human embryonic stem cells (hESCs) and human induced pluripotent stem cells (hiPSCs) [106]. hESCs are derived from the inner cell mass (ICM) of embryos and have the ability to differentiate into three lineages including ectoderm, mesoderm, and endoderm [107]. hiPSCs are generated by reprogramming somatic cells to an embryonic-like state [108]. Both hESCs and hiPSCs can differentiate into all cell types in the body, while iPSCs offer personalized genetic information and pathological phenotypes [109]. Pietilainen et al. generated the co-culture system of hPSC-derived astrocytes and neurons, demonstrating that astrocytes had an increased expression of genes encoding synaptic cell adhesion molecules, which significantly influenced the expression of synaptic gene programs in neurons [110]. Drager et al. utilized the hiPSC-derived microglia model, exhibiting robust phagocytic activity, responding to inflammatory stimuli, and displaying morphological changes characteristic of activated microglia upon bacterial-derived lipopolysaccharide stimulation [111]. Stebbins et al. developed hPSC-derived pericyte-like cells, which can self-assemble with ECs and significantly enhance BBB properties in BMECs through barrier tightening and the reduction in transcytosis [112].

### 3.2. 3D Organoids

Brain organoids are self-aggregated 3D brain models generated from hPSCs [113]. Brain organoids can recapitulate key structural and functional aspects of real brain tissues, including the cellular migration that occurs during brain development [114,115]. They have been used to model various neurological disorders, such as Zika virus infection, and have provided insights into the genetic mechanisms and pathogenic pathways of these diseases. [116,117] They offer advantages over animal models and traditional 2D cell models, allowing valuable information on live and functional tissues.

#### 3.2.1. Unguided (Cerebral Organoid)

Unguided brain organoids are developed with the minimized influence of external patterning factors [118]. These brain organoids rely on the intrinsic differentiation capacities of hPSC aggregates to spontaneously form 3D structures containing various brain-like regions and corticogenesis. The process of morphogenesis in unguided brain organoids is self-directed, which can lead to a diverse range of cell types and cytoarchitectures similar to those found in the human embryonic brain [119]. Unguided brain organoids are used to study the complex processes of human brain development, such as progenitor proliferation and neuronal subtype distribution. However, the lack of external guidance can result in variability between organoid samples [10,120]. Lancaster et al. developed the first unguided brain organoid, termed cerebral organoids, from hPSCs to investigate the key mechanism of microcephaly by modeling the cerebral cortex containing progenitor cells, their structural orientation, and mature neuronal differentiation. The organoids formed large, continuous neuroepithelia surrounding a fluid-filled cavity, with apical localization of neural-specific N-cadherin, indicating early brain development stages. It was observed that a heterozygous mutation in the CDK5RAP2 gene leads to premature neural differentiation and microcephaly utilizing primary microcephaly patient-derived organoids [116]. Matrigel is used to provide structural support, promoting the continuity and proper orientation of the neuroepithelium [118]. These organoids exhibit interconnected ventral and dorsal areas, revealing the presence of forebrain organizing centers, which are crucial for dorso-ventral patterning [121]. Due to the lack of blood supply, which restricts oxygen and nutrient availability, brain organoids have been limited with hypoxia-driven cell apoptosis and neuronal maturation. Giandomenico et.al. reported the air–liquid interface (ALI-COs) to improve survival, morphology, and functional output for cerebral organoids. Markers such as SMI312 for axons and MAP2 for dendrites have been detected, indicating successful neuronal differentiation and tract formation [122]. Pellegrini et al. designed the Choroid plexus (ChP) organoids by utilizing the dorsalizing factor BMP4 and the WNT-activator compound, CHIR99021, to induce ChP fate in cerebral organoids, which actively secrete a colorless fluid and closely mimic the properties of cerebrospinal fluid (CSF) [123]. Bagley et al. developed the fused cerebral organoids model, presenting the interactions between different brain regions and the migratory dynamics of cortical interneurons [124]. Despite the variability across different batches, unguided brain organoids can recapitulate aspects of brain complexity and contain a broad diversity of cells that are related to endogenous classes, including cells from the cerebral cortex and the retina [125,126].

#### 3.2.2. Regionalized (Cortical Organoids, Assembloids)

Guided organoids, termed regionalized brain organoids, involve the supplementation of specific growth factors and other signaling molecules that mimic the developmental cues of the human brain [119]. Guided organoids are cultivated using protocols that steer the differentiation of cells into particular cortical layer-specific neuronal cells, functional synapses, glial cell types, and brain regions (e.g., ventral zone) [127]. Pasca et al. developed human cortical spheroids (hCSs), which can resemble the cerebral cortex [128]. The floating hCSs were generated from hPSC colonies cultured in nonadherent conditions without extracellular scaffolding. The expression of deep-layer markers (e.g., TBR1 and CTIP2) and superficial-layer markers (e.g., SATB2 and BRN2) indicate that the hCSs contain neurons from both deep and superficial cortical layers, which are organized in a laminated structure similar to the natural cerebral cortex [128]. These neurons are electrophysiologically mature, meaning they can fire and transmit electrical signals like those in the actual human brain. Additionally, hCSs exhibit spontaneous activity and form functional synapses, which are crucial for communication between neurons [129]. Jo et al. developed human midbrain-like organoids (hMLOs), which have the ability to produce human neuromelanin, a unique characteristic of the human midbrain [130]. These organoids are capable of secreting dopamine, a critical neurotransmitter associated with motor control and reward pathways, and this feature is particularly relevant for modeling diseases like Parkinson‘s disease since the key factor of such a disease is the degeneration of nigral dopaminergic neurons [130,131].

These organoids can also be used to create assembloids by fusing spheroids from different brain regions, termed assembloids, allowing researchers to study inter-regional interactions [125]. Assembloids are sophisticated constructs formed by physically fusing the different brain region-specific organoids to model the interactions and connectivity between various brain areas [119]. Also, assembloids allow researchers to observe the migration of interneurons and other cellular interactions that occur between distinct brain areas [10]. Birey et al. demonstrated the in vitro assembly of human forebrain spheroids that recapitulate the migration and functional integration of GABAergic interneurons with glutamatergic neuron circuits [115]. hCSs and human subpallium spheroids (hSSs) are independently generated through a differentiation process that involves modulation of the WNT and SHH pathway and then cultured together to allow for migration and integration [115]. The findings also suggest that abnormalities in interneuron migration could contribute to the pathophysiology of Timothy syndrome (TS), a neurodevelopmental disorder, and that these migration defects can be pharmacologically rescued [132,133]. By patterning spheroids into specific brain regions (e.g., hCSs and hSSs), Sloan et al. then physically brought them together in a co-culture system to promote fusion. The optimal window for fusion is between days 30 to 90 of differentiation, beyond which the efficiency of spheroid fusion may decrease. The model successfully developed the dorsal and ventral forebrain, capturing the migration and integration of GABAergic neurons into the cerebral cortex [114]. Miura et al. developed cortico-striatal assembloids modeling the functional connectivity of human brain circuits, showing increased intrinsic excitability and synaptic activity in striatal neurons when assembled with cortical spheroids [134]. These assembloids can recapitulate disease-specific neural activity, which exhibits altered calcium signaling and network synchronization [134]. Additionally, they generated functional human 3D cortico-motor assembloids, providing evidence that corticofugal neurons can project to and connect with spinal spheroids, and that spinal-derived motor neurons can form connections with muscle tissue, enabling the contraction of 3D muscle upon stimulation [135,136]. They are particularly useful for studying neurodevelopmental processes and diseases that involve multiple brain regions.

#### 3.2.3. Bioengineered

In addition to the traditional organoids, novel bioengineered organoids combined with biomaterials and genetic engineering modification strategies have been developed to address the limitations of conventional brain organoid models. Lancaster et al. invented the microfilament-based methodology to serve as scaffolds organized to enhance neuroectoderm formation and cortical development by facilitating guided self-organization through neuroepithelium elongation [137]. Natural materials (e.g., sea sponge) and synthetic biocompatible materials (e.g., polylactide-co-glycolide (PLGA)) are also tested; however, PLGA polymers showed better results maintaining dense cell composition while promoting elongated morphologies of the neural ectoderm [137]. Cho et al. created a microenvironment, similar to the in vivo state, by developing a human-brain-derived decellularized extracellular matrix (BEM) and microfluidic device in the cultivation process [138]. Compared to the non-specific matrix scaffold (i.e., Matrigel), BEM provided improved neural differentiation and corticogenesis through brain-mimetic biochemical niche [138]. Moreover, the microfluidic devices fabricated by the soft lithography technique are utilized to mimic a fluid flow in the cerebrospinal and interstitial spaces. The results indicate that microfluidic systems can improve the oxygen supply and nutrient/waste exchange, resulting in a significant reduction in cell death throughout the organoid structure [138]. Cakir et al. genetically engineered vascularized human cortical organoids (vhCOs) by inducing the expression of the transcription factor ETV2 in hESCs, which leads to the differentiation of these cells into ECs. The optimal formation of vascular-like structures within the organoids was achieved by adjusting the ratio of ETV2-expressing cells to 20% and the timing of their induction at day 18. The expressed EC markers such as CDH5, CD31, KDR, TEK, vWF, and CD34, show the presence of vascular-like structures, which is absent in traditional organoids [139].

### 3.3. Organ-on-Chip

Brain-on-a-chip (BoC) is an emerging technology that aims to mimic the structure and function of the human brain in a microfluidic device. It combines the principles of microfluidics, tissue engineering, and stem cell biology to create a platform for studying the brain in a controlled fluidic condition and realistic environment. Organ-on-chip systems allow the diverse microphysiology of the human brain including the BBB, inflammatory system, and multi-organ communications [140,141].

#### 3.3.1. Blood–Brain Barrier

The BBB is a crucial structure between the blood and the brain [142]. It regulates the transport of molecules, protects against pathogens, and thus is a major hurdle in efficient drug delivery to the brain [143]. Traditional in vitro models of the BBB have limitations in replicating the complex anatomical and physiological features of the BBB. However, recent advancements in organ-on-chip technology have led to the development of BBB-on-a-chip models. These models use microfluidic devices to recreate the BBB microenvironment and cell–cell interactions more accurately by incorporating BMECs, astrocytes, pericytes, and various neural cells to mimic the structure and function of the BBB [144]. BBB-on-a-chip models offer several advantages, including the ability to study disease pathophysiology, screen drug candidates, and predict drug transport efficacy [145]. Griep et al. developed the BBB-on-a-chip model with two channels, each measuring 1 cm in length, 500 µm in width, and 100 µm in depth, separated by a transwell polycarbonate membrane with a pore size of 0.4 µm. Platinum (Pt) electrodes are integrated into grooves on the chip to measure electrical impedance across the membrane. Mechanical stimulation through shear stress significantly enhanced barrier tightness, as reflected by a threefold increase in TEER values, while exposure to tumor necrosis factor-alpha (TNF-α), a pro-inflammatory cytokine, resulted in a tenfold decrease in TEER, demonstrating the model’s capability to biochemically modulate barrier function and mimic inflammatory conditions relevant to neurodegenerative diseases [146]. Yu et al. presented a novel 3D microfluidic model of the BBB by a pump-free strategy that uses gravity as the driving force and a paper-based flow resistor to regulate resistance, thus maintaining a flow rate that is physiologically relevant to brain capillaries [147]. The model showed a decrease in occludin gene expression upon exposure to TNF-α, indicating a compromise in the tight junctions, and an increased production of IL6 and CINC1, cytokines known to mediate neuroinflammation [147].

#### 3.3.2. Neuroinflammation

Neuroinflammation refers to inflammation that occurs in the CNS as a response to various insults or injuries [148]. It involves the activation of immune cells (e.g., microglia, astrocytes) and the release of pro-inflammatory molecules (e.g., cytokines, chemokines) triggered by a variety of factors, such as infection [149]. The inflammatory response in the CNS acts as a double-edged sword role, with both beneficial and detrimental effects, as it can contribute to tissue repair and the clearance of pathogens, but it can also lead to neuronal damage and dysfunction [10,150]. Pediaditakis et al. developed a microengineered Brain-Chip designed to model neuroinflammation in humans, successfully captured complex functional interactions within the neurovascular unit, and demonstrated in vivo-like gene expression signatures. Upon exposure to pro-inflammatory cytokines, the Brain-Chip reproduces key features of neuroinflammation, including markers of glial activation and increased barrier permeability, providing a valuable tool for studying the communication between the CNS and peripheral organs [149]. Herland et al. also developed a microengineered BBB-on-chip model to study neurovascular inflammation by incorporating a cylindrical collagen gel with a central hollow lumen, where primary human brain microvascular endothelial cells are cultured on the gel’s inner surface, and medium flow is maintained through the lumen. The model demonstrates enhanced secretion profiles for inflammatory markers like granulocyte colony-stimulating factor (G-CSF) and interleukin-6 (IL-6) upon stimulation with TNF-α [151].

#### 3.3.3. Multi-Organ Crosstalk

The gut–brain axis refers to the bidirectional communication between the gut and the brain. It involves immune, endocrine, and metabolic signaling mechanisms of the brain and gut microbiota [152]. The gut microbiota can affect CNS function through the synthesis of neurotransmitters and neuromodulators, as well as by modulating the function of intestinal cells [153]. Disturbances in the gut–brain axis have been implicated in neurodegenerative disorders such as AD [153,154,155]. Kim et al. developed a modular microfluidic chip that mimics the gut–brain axis by co-culturing gut epithelial cells and BMECs to create a model of the gut epithelial barrier and the BBB. The chip is designed to be easily assembled and disassembled, with channels that provide fluidic flow and porous membranes that support cell culture, replicating the barrier functions of the corresponding tissues [154]. The lung–brain axis refers to the bidirectional interaction between the lung and the brain through signals and molecules exchange, in homeostatic and pathological condition [156]. The lung is a critical organ for oxygenation and the removal of waste products, and it also produces various bioactive substances that can influence brain function [157]. On the other hand, the brain can modulate lung function through neural pathways and the release of neurotransmitters [158]. The lung–brain axis has been implicated in various conditions, including respiratory diseases, neurodegenerative disorders, and mental health disorders [156,157,158]. Wang et al. designed an integrated microphysiological system (MPS), utilizing a lung alveolus chip infected with the SARS-CoV-2 virus and a connected BBB chip to assess subsequent brain microvascular damage. A significant increase in BBB permeability was observed, with the disruption of adherents and tight junctions in BMECS, suggesting that the neurological alterations observed in the model are likely due to indirect effects following pulmonary infection, rather than a direct viral invasion of the brain [7].

### 3.4. Bioengineered Tissue Models

Traditional tissue engineering approaches have fallen short of mimicking the intricate organization of living tissues. Through strategies involving the co-emergence of diverse cellular sources, biomaterials, bioprinting, and other devices can be improved to form complex patterns.

There were advances in biomaterials, including natural materials (e.g., collagen, gelatin, silk, alginate, cellulose) and synthetic polymers (e.g., polyglycolic acid; PGA, polylactic acid; PLA, and poly(lactic-co-glycolic) acid; PLGA) [159,160]. Shin et al. reported on 3D electroconductive hyaluronic acid (HA) hydrogels, integrated with carbon nanotubes (CNTs) and polypyrrole (PPy) to enhance the neurogenesis of human neural stem/progenitor cells (hNSPCs). They provided a dynamic and biocompatible extracellular matrix that promotes neuronal differentiation and improves electrophysiological functionality [161]. These hydrogels provide a conducive 3D environment that mimics in vivo conditions, promoting neurogenesis by upregulating calcium channel expression and increasing intracellular calcium influx [161].

Roth et al. developed a bioprinting platform called Spatially Patterned Organoid Transfer (SPOT), which enables the spatially controlled construction of assembloids from organoid building blocks (OBBs) [162]. The platform utilizes magnetic nanoparticles (MNPs) to temporarily coat and maneuver OBBs, allowing for precise spatial control during the construction of assembloids without causing structural deformation [162]. Park et al. developed multilayered cerebrovascular conduits (MCCs) using a hybrid cells-laden brain-derived extracellular matrix (BdECM) bio-ink, which includes human BMECs, human brain vascular pericytes, and neural progenitor cells. This bioprinted model features perfusable blood vessels with controlled geometries [163]. Jin et al. overcame the challenges associated with handling mature neurons, which are sensitive to physical stress and damage during the 3D printing process, by printing neural progenitors that differentiate into mature neurons post-printing [164]. The research demonstrated the application of 3D-printed neural progenitors to integrate and differentiate within an ex vivo lesioned brain slice for brain repair strategies [164]. Grebenyuk et al. created the soft microfluidic grids with capillary-like tubing by printing a custom hydrophilic photopolymer based on polyethylene glycol diacrylate (PEGDA) on a hard plastic base to form the tight seal [165].

Fattah et al. applied mechanical forces on human neural tube organoids (hNTOs) to enhance their growth and patterning [166]. They used equibiaxial and uniaxial stretching devices to apply controlled mechanical strains to elastomeric membranes containing the hNTOs. Finite Element Analysis (FEA) was used to simulate the mechanical environment and stresses experienced by the organoids, suggesting that incorporating mechanical actuation in organoid culture systems can lead to more reproducible and programmable tissue models [166]. The authors also used the novel 3D soft microfluidic strategy to create synthetic capillary-scale vessels, enabling the perfusion of large tissue constructs and overcoming the limitations of previous in vitro tissue engineering approaches [165]. Rifes et al. introduced a novel method called microfluidic-controlled stem cell regionalization (MiSTR) to model early human neural tube development using hESCs by employing signaling gradients, particularly WNT, to mimic the developmental patterning of in vivo and created neural tissue with rostral-to-caudal neural axis [167].

**Table 1:** Advantages and disadvantages of different in vitro human brain models.

In Vitro Model		Advantages	Disadvantages	Citations
2D Monolayer Cultures	Primary Cells	Properties of native brain tissues.	Challenges of accessibility, limited proliferation, inconsistent results, and lower growth rates.	Danz et al. [94]
	Immortal Cell Lines	Indefinite proliferation, robust, and easy to manipulate.	Alter physiological properties due to genetic modification.	He et al. [97], Chua et al. [98], Zhang et al. [99], Slanzi et al. [100]
	Stem Cells (NSCs, hPSCs)	Differentiation into various specialized cell types, personalized genetic information (hiPSCs).	Complicated cultural conditions, and potential variability in differentiation outcomes.	Pietilainen et al. [110], Drager et al. [111], Stebbins et al. [112]
3D Organoids	Unguided Brain Organoids	Spontaneous formation of diverse brain regions, suitable model for studying brain development.	Variability between samples, lack of external guidance.	Lancaster et al. [116], Giandomenico et al. [122], Pellegrini et al. [123], Bagley et al. [124]
	Regionalized Organoids	Specific growth factors combined for region-specific differentiation, form functional synapses, and model disease-specific neural activity.	Skillset of precise control of differentiation protocols.	Pasca et al. [128], Jo et al. [130]
	Assembloids	Recapitulation of interactions and connectivity between various brain regions, involving multiple brain regions.	Batch variation between samples, inconsistent producibility, and unstable long-term cultures.	Birey et al. [115], Sloan et al. [114], Miura et al. [134]
	Bioengineered Organoids	Improved 3D structural support, and complex microenvironment modeling.	Complex to develop, limited nutrient and waste exchange.	Lancaster et al. [137], Cho et al. [138], Cakir et al. [139]
Brain-on-a-Chip (BoC)	BBB-on-a-Chip	Functional BBB microenvironment, useful for drug testing.	Limitations in replicating the entire BBB functionality.	Griep et al. [146], Yu et al. [147]
	Neuroinflammation Models	Residual and peripheral inflammatory responses.	Requires precise manipulation, variability due to complex mixture of different cells.	Pediaditakis et al. [149], Herland et al. [151]
	Multi-Organ Chip Models	Mimics organ crosstalk including gut–brain and lung–brain interactions.	Complex and high-cost to fabricate, limited ability to fully emulate the systemic interactions.	Kim et al. [154], Wang et al. [7]
Bioengineered Tissue Models	Biomaterials	Enhanced neurogenesis and neural differentiation, variable choice of materials, reduced cell death, and vascularization in brain models.	Potential biocompatibility issues, variation due to manipulation of biomaterials.	Shin et al. [161]
	Bioprinting	Precise spatial control, improved integration of multiple cell types and materials, allows for high reproducibility and scalability.	Technical complexity, high cost of equipment and materials, potential issues with cell viability and functionality post-printing, and requires specialized knowledge and skills for operation.	Roth et. al. [162], Park et al. [163], Jin et al. [164], Grebenyuk et al. [165]

## 4. Application of Infection Model for Neurodegenerative Diseases and Neurological Disorder

**Figure 3:**
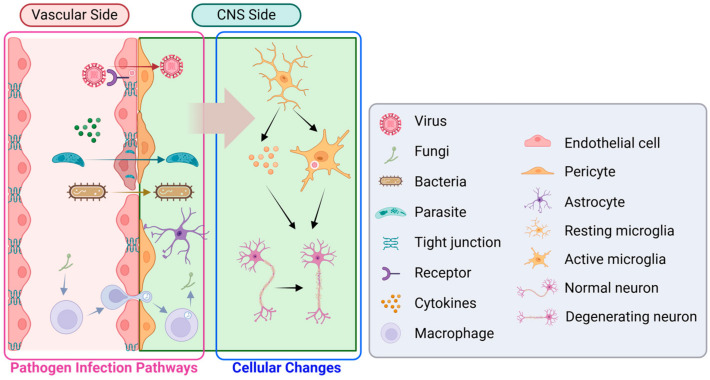
Pathogen infection in the BBB and subsequent neuroinflammation in CNS. Pathogen infection can lead to BBB disruption or penetration via transcellular, paracellular, and Trojan horse strategies. The subsequent responses in the CNS include astrocytes and microglia recruitment, activation., phenotypical changes, and the secretion of cytokines, causing the neurons to degenerate as local immune reactions.

Recent advancements in the in vitro models of human brain have become increasingly important to study the effects of viruses, fungi, bacteria, and parasites on brain function, size, and cytoarchitecture (Table 2), and their further development of therapeutics for brain infectious diseases (Figure 3).

### 4.1. Virus

In vitro brain models have provided insights into the vulnerability of neural lineage to viral infections and the mechanisms by which these infections lead to neurodegeneration or neurological disorder including microcephaly. In addition to the impact on the brain, the mechanism of virus penetration of the BBB demonstrated the ability of pathogens (e.g., SARS-CoV-2) to cross the BBB by disrupting its integrity [168].

#### 4.1.1. ZIKV

In vitro models facilitate ZIKV research, specifically the brain, which is a major target of ZIKV infection. In particular, brain organoids enable a more accurate pathological study than traditional 2D cell cultures by offering a 3D structure that mimics the complexity of human brain tissues [169]. Xu et al. developed region-specific organoids to study the ZIKV infection. These organoids comprise distinct ventricular zone (VZ) and subventricular zone (SVZ) layers, mimicking the composition and organization of the developing human fetal brain [117]. Upon ZIKV infection, the model exhibits characteristics similar to fetal microcephaly, such as the thinning of the VZ layer and reduced organoid size. Remarkably, treatment with enoxacin, an RNAi enhancer, effectively prevented ZIKV infection and the associated damage in these brain organoids, maintaining normal cell proliferation and organized cell layers [117]. Garcez et al. used hiPSC-derived brain organoids and found that ZIKV-infected organoids displayed morphological abnormalities and cell detachment, while the growth area of ZIKV-exposed organoids was significantly reduced compared to controls. This suggests that ZIKV disrupts neurogenesis, which could explain the link between ZIKV infection and congenital brain malformations like microcephaly [170]. Alimonti et al. utilized a 2D transwell system integrated with brain endothelial cells (BECs), neural progenitor cells, and mature neurons to study the infection route of ZIKV in the brain. The model has demonstrated that ZIKV can infect BECs and cross the human BBB without compromising the integrity of tight junctions, by identifying the expression of AXL, a receptor implicated in the entry of ZIKV into host cells, and the transendothelial electrical resistance (TEER) value showing that paracellular permeability remained unchanged [171].

#### 4.1.2. SARS-CoV-2

The SARS-CoV-2 virus could cause neurological dysfunction, through direct infection or secondary effects like inflammation or hypoxia [172,173]. Glial cells, including astrocytes and microglia, express the angiotensin-converting enzyme 2 (ACE2) receptor, which SARS-CoV-2 uses to enter and infect the brain. During severe COVID-19, the increase in circulating chemokines and interleukins can compromise the BBB, allowing these inflammatory mediators to affect glial cells [174]. While SARS-CoV-2 can be detected in the brain, it predominantly affects vascular and immune cells rather than directly infecting neurons. The neurological symptoms, such as anosmia, are primarily due to inflammatory processes involving microglial activation and subsequent neuronal damage [175]. Buzhdygan et al. showed that the spike protein of the SARS-CoV-2 virus can alter barrier function in both 2D static and 3D microfluidic in vitro BBB models [168]. The study provided the first evidence that the SARS-CoV-2 spike protein can trigger a proinflammatory response in human BMECs, which may lead to an altered state of BBB function. Additionally, the research explored the expression of ACE2 receptor in the cerebral vasculature and its upregulation in pathological conditions like hypertension and dementia [168]. Wang et al. used a lung–brain microphysiological system (MPS) that simulates the human BBB and alveolar–capillary barrier to study the effects of SARS-CoV-2 on the brain [7]. The lung–brain MPS demonstrated that direct exposure of the BBB to SARS-CoV-2 caused only mild changes. However, they suggest that the virus could indirectly induce more severe BBB injuries and brain inflammation from systemic inflammation, rather than direct viral invasion of the brain through the bloodstream [7]. Ramani et al. revealed that SARS-CoV-2 prefers mature neuronal cell types, as indicated by higher infection rates in Day-60 brain organoids compared to Day-15 organoids [176]. The study also shows altered Tau protein distribution, hyperphosphorylation, and signs of neuronal death following SARS-CoV-2 exposure [176]. Jacob et al. developed hiPSC-derived monolayer neural cells and region-specific brain organoids to study SARS-CoV-2 neurotropism [177]. The choroid plexus organoids derived from these stem cells have shown high susceptibility to SARS-CoV-2, leading to productive infection and increased cell death [177]. Martínez-Mármo et al. specifically investigated the virus-induced fusion between neurons and neurons or glial cells utilizing 3D brain organoids by comparing those findings with other animal models [178]. The study demonstrates that the SARS-CoV-2 spike (S) protein causes the formation of multicellular syncytia, which are clusters of interconnected neurons and found that such fusion events severely compromised neuronal activity, as demonstrated by disruptions in calcium signaling within the neurons, suggesting that the neurological symptoms associated with COVID-19 [178]. Aguado et al. use the human brain organoids to induce cellular senescence and mimic the SARS-CoV-2 infection process [179]. Senolytic therapy, which targets and eliminates senescent cells, has been shown to reduce inflammation and rejuvenate aging markers in the brain [180]. Additionally, this therapeutic approach can block viral replication and prevent cellular senescence in brain organoids infected with SARS-CoV-2 [179]. Wang et al. develop a pericyte-containing cortical organoids model to mimic the NVU and study SARS-CoV-2 neuropathology [181]. The pericyte-containing cortical organoids demonstrate increased susceptibility to SARS-CoV-2 infection compared to traditional cortical brain organoids, with a significant increase in viral RNA and nucleocapsid protein in infected cells [181].

#### 4.1.3. Other Viruses

Fares et al. developed a 2D co-culture model to understand how tick-borne encephalitis virus (TBEV) affects human neurons and astrocytes [182]. Astrocytes exhibit limited susceptibility to infection and display astrogliosis, which is a response to stress. Neuronal death occurred as early as 72 h post-infection, and there was a significant loss of neurites, which are essential for neuron function. The model also demonstrated that astrocytes exert a protective effect on neurons, and while astrocytes were also affected by the virus, their survival was less impacted compared to neurons [182]. Reis et al. have developed a 3D human brain organoid showing an increased inflammatory response similar to that observed in HIV-infected individuals, with higher levels of TNF-α and interleukin-1 beta (IL-1β), the key markers of the neuroinflammatory environment observed in HIV-infected brains [183]. The infected microglia within the organoid released neurotoxic factors, leading to a reduction in the number of live neurons and an increase in dead cells. There was a marked decrease in the expression of the neuronal marker bIII-Tubulin, suggesting neuronal loss, and an increase in the astrocyte marker GFAP, indicating astrocytosis. The synaptic integrity was compromised, with a significant reduction in the pre- and post-synaptic markers Synaptophysin and PSD-95, respectively [183]. By growing hiPSC-derived brain organoids in a spinning bioreactor to enhance nutrient and oxygen exchange, Zhang et al. found that the brain organoids exhibited a decrease in overall size after infection with influenza viruses, similar to the effects observed with Zika virus infection [184]. Specifically, the organoids showed widespread infection across multiple cell types, with a preference for infecting neurons, when infected with the H1N1-WSN strain, leading to apoptosis in both neurons and NSCs, but not in astrocytes [184]. Cairns et al. have developed a 3D bioengineered human brain model encapsulated in laminin and incorporated collagen type I [185]. After being infected with a low level of herpes simplex virus type 1 (HSV-1) for one week, the model showed key pathological symptoms of AD such as amyloid plaque-like structure formation; moreover, gliosis and neuroinflammation reactions are observed with evidence of the upregulation of GFAP and TNF expression [185]. Progressive multifocal leukoencephalopathy (PML) is a serious brain condition often seen in people with weakened immune systems, caused by John Cunningham virus (JCV) infection. Barreras et al. have created an iPSC-derived 3D brain organoid model to study the JCV infection and the development of PML. The organoids are exposed to the JCV-MAD4 strain or cerebrospinal fluid from a PML patient to initiate infection. Immunocytochemical studies revealed viral antigens within the oligodendrocytes and astrocytes, but not within neurons [186]. D’Aiuto et al. explored the scaffold-free 3D human neuronal cultures infected with HSV-1, and further assessed the efficacy of the antiviral drug, acyclovir, showing that the drug was effective in inhibiting viral replication within the 3D cultures [187].

### 4.2. Fungi

Koutsouras et al. cultured microglia using BV-2 cell lines to study the interactions between *Cryptococcus neoformans* and host cells [188]. After infection in the in vitro model, microglial cells undergo morphological changes, increase the expression of MHC II, and enhance their phagocytic function in response to fungal pathogens [188]. Kim et al. developed a human-neurovascular-unit (hNVU)-on-a-chip (hNVU chip) that models the BBB and allows for the study of pathogen penetration, specifically *Cryptococcus neoformans*, the leading cause of fungal meningitis [189]. The hNVU chip includes human NSCs, BMECs, and brain vascular pericytes co-cultured in a microfluidic device, showing that *Cryptococcus neoformans* can get through the BBB without breaking the tight connections between cells. The secretion levels of certain paracrine factors (e.g., PTX3, TSP-1, IL-8) also increased significantly, indicating a host response to the fungal infection. The research provides insights into the neurotropic behavior of *Cryptococcus neoformans* and identifies specific cryptococcal factors that facilitate BBB crossing, which could be targets for therapeutic intervention [189].

### 4.3. Bacteria

Different bacteria have distinct mechanisms for disrupting the BBB and invading the CNS, which is critical for bacterial pathogenicity, particularly in meningitis. Bacteria like *Staphylococcus aureus* (*SA*) and *Neisseria meningitides* (*NmA*) manipulate host cell receptors and junctional proteins to compromise the BBB integrity [190,191]. McLoughlin et al. use the primary non-transformed human BMECs to simulate the BBB and study the effects of SA infection [192]. The model is infected by both formaldehyde-fixed SA, which maintains bacterial structure without the metabolic activity of live SA, showing that SA can adhere to human BMECs, elevate paracellular permeability, and induce a dose-dependent release of cytokines and chemokines. The result showed a reduced expression of interendothelial junction proteins (i.e., VE-Cadherin, claudin-5, and ZO-1), which are crucial for maintaining BBB integrity [192]. Endres et al. develop an in vitro model of the human meningeal blood-cerebrospinal fluid barrier, using BMECs derived from iPSCs in co-culture with leptomeningeal cells (LMCs) from meningioma biopsies on permeable transwell, to study (*NmA*) infection. After infection, the model showed considerable amounts of bacteria adhering to the BECs, with a smaller number of bacteria found inside the cells. Despite the presence of bacteria, the barrier integrity remained high for the first 24 h post-infection, suggesting that the bacteria might be crossing the barrier through a transcellular route. However, at later time points, there was a deterioration of barrier properties, including a loss of TEER and reduced expression of cell-junction components indicating that prolonged infection can compromise the barrier, potentially allowing for paracellular crossing of the bacteria [193].

### 4.4. Parasite

*T. gondii* is a parasite that infects around one-third of the global population, leading to chronic infections where the parasite forms cysts in brain neurons. hiPSC-derived neurons and induced neurons (iNs) were used for studying the effects of *T. gondii* on neurons and neurological functions [194,195]. Seo et al. demonstrated that human cerebral organoids can be infected by *T. gondii*, with the parasite completing its asexual life cycle within the organoids [196]. The model has shown that *T. gondii* can infect various brain cell types within the organoids, such as neurons, astrocytes, and oligodendrocytes. Transcriptomic analysis revealed that the organoids activate a type I interferon immune response against the infection, and *T. gondii* itself shows changes in its transcriptome related to invasion and replication [196]. Leite et al. used 3D brain spheroids to study the morphological and biochemical impacts of *T. gondii* infection on human neural cells [197]. The parasite was able to infect and proliferate within the spheroids, leading to the formation of cysts and causing neural cell death. Morphological alterations were observed, including degenerated cells on the surface of the spheroids and a reduction in their size. Additionally, there was an increase in the release of lactate dehydrogenase (LDH), indicating cellular damage [197]. Coronado-Velazquez et al. developed an in vitro model of the BBB to study the interaction between *Naegleria fowleri*, a pathogenic amoeba, and the ECs, using primary rat BMECs. After being infected with *Naegleria fowleri*, the amoeba caused a decrease in TEER, indicating a compromise in the integrity of the endothelial barrier, and triggered the degradation of tight junction proteins (i.e., claudin-5, occludin, ZO-1) which are crucial for maintaining the barrier function. These changes suggest that *Naegleria fowleri* disrupts the BBB, allowing peripheral blood cells to access the CNS, which could lead to inflammation and further complications associated with primary amoebic meningoencephalitis [198].

**Table 2:** Application of infection model for neurodegenerative diseases and neurological disorder.

Pathogens		Cell Resources	In Vitro Models	Insights	Citations
Virus	Zika Virus (ZIKV)	Neural progenitor cells, mature neurons, BMECs	Region-specific brain organoids, hiPSC-derived brain organoids, 2D transwell system	ZIKV infection leads to microcephaly-like symptoms, disrupts neurogenesis, and can cross the BBB without compromising the integrity of tight junctions. Enoxacin treatment prevents ZIKV-associated damage [117,170,171].	Xu et al. [117], Garcez et al. [170], Alimonti et al. [171].
	SARS-CoV-2	Astrocytes, microglia, neurons, BMECs	2D static and 3D microfluidic BBB models, lung–brain MPS, brain organoids, hiPSC-derived monolayer neural cells, choroid plexus organoids, pericyte-containing cortical organoids	SARS-CoV-2 affects brain function through direct infection and secondary effects. It primarily affects vascular and immune cells, leading to neurological symptoms through inflammatory processes. The spike protein disrupts BBB integrity [176,177,178,179].	Buzhdygan et al. [168], Wang et al. [7], Ramani et al. [176], Jacob et al. [177], Martínez-Mármo et al. [178], Aguado et al. [179], Wang et al. [181].
	Tick-borne encephalitis virus (TBEV)	Neurons, astrocytes	2D co-culture model	TBEV leads to neuronal death and neurite loss, with astrocytes showing limited infection but exhibiting astrogliosis to protect neurons [182].	Fares et al. [182].
	Human immunodeficiency virus (HIV)	Neurons, microglia, astrocytes	3D human brain organoid	HIV infection leads to increased inflammatory response, neuronal loss, astrocytosis, and compromised synaptic integrity [183].	Reis et al. [183].
	Influenza virus (H1N1-WSN strain)	Neurons, NSCs, astrocytes	hiPSC-derived brain organoids in a spinning bioreactor	The virus causes widespread infection, apoptosis in neurons and NSCs, and reduced organoid size [184].	Zhang et al. [184].
	Herpes simplex virus type 1 (HSV-1)	Neurons, glial cells	3D bioengineered human brain model, scaffold-free 3D human neuronal cultures	HSV-1 infection shows amyloid plaque-like structures, gliosis, and neuroinflammation, and is effectively inhibited by acyclovir [185,187].	Cairns et al. [185], D’Aiuto et al. [187].
	John Cunningham virus (JCV)	Oligodendrocytes, astrocytes, neurons	iPSC-derived 3D brain organoid	JCV infects oligodendrocytes and astrocytes, not neurons, leading to PML [186].	Barreras et al. [186].
Fungi	*Cryptococcus neoformans*	Murine BV-2 cell lines, microglia, NSCs, BMECs, brain vascular pericytes	2D static model, human-neurovascular-unit-on-a-chip (hNVU chip)	The fungus causes microglial activation, and phagocytosis, and crosses the BBB without disrupting tight junctions [188,189].	Koutsouras et al. [188], Kim et al. [189].
Bacteria	*Staphylococcus aureus (SA)*	BMECs	2D static BBB model	SA infection increases paracellular permeability and cytokine release, reducing the expression of interendothelial junction proteins [192].	McLoughlin et al. [192].
	*Neisseria meningitidis*	BMECs, leptomeningeal cells	iPSC-derived BMECs co-cultured with leptomeningeal cells on transwell system	The bacteria adhere to BMECs, cross the barrier transcellularly, and compromise barrier integrity over time [193].	Endres et al. [193].
Parasite	*Toxoplasma gondii (T. gondii)*	Neurons, astrocytes, oligodendrocytes	Human cerebral organoids, 3D brain spheroids	T. gondii infects various brain cells, causing neural cell death, size reduction in organoids, and cellular damage [197].	Seo et al. [196], Leite et al. [197].
	*Naegleria fowleri*	Rat BMECs	2D static BBB model	The amoeba decreases TEER, degrades tight junction proteins, and disrupts the BBB, leading to potential CNS inflammation [198].	Coronado-Velazquez et al. [198].

## 5. Conclusions and Future Directions

In summary, this review highlights the diverse cellular composition of the human brain and the development of various in vitro brain models to study pathogen infection-related neurodegeneration. The human brain is a highly complex organ composed of various populations of neurons, astrocytes, oligodendrocytes, microglia, and surrounding systems including BBB, CSF, and neuroimmune systems. These cells work in a coordinated manner to maintain brain homeostasis and function. The advancements in cell and tissue engineering technologies have enabled the generation of increasingly sophisticated in vitro human brain models, including 2D monolayer cultures, 3D organoids, organ-on-chips, and bioengineered tissue models. These models aim to recapitulate the cellular diversity, structural organization, and functional properties of the native human brain, making them valuable tools for studying pathogen infection and its impact on the nervous system. The models have facilitated a deeper understanding of how different pathogens, such as viruses, bacteria, and parasites, can directly or indirectly affect brain cell functions and lead to neurological complications. Furthermore, these models have enabled the discovery of diagnostic markers and the identification of potential therapeutic targets for addressing pathogen infection-related neurodegeneration. 

Despite recent progress of the human brain model and its application to investigate the pathogen–host responses, they are still limited by the less diverse cellular population, incomplete microenvironment, and lack of systemic construction at a tissue scale. Therefore, to reveal the underlying mechanism of the pathogen infection and host reaction in relation to neurodegeneration and neurological disorder, technological advances in stem cell biology and tissue engineering are highly required for the following specific studies. A 3D bioprinting strategy has been optimized to fabricate neurospheroid-laden fibers within an astrocyte-laden support hydrogel, aiming to mimic the brain’s stiffness and facilitate the growth and differentiation of NSCs and astrocytes [199]. Techniques such as inkjet bioprinting and extrusion-based methods are employed to deposit bio-inks, which are designed to support cell growth and mimic the extracellular matrix [200]. A 4D bioprinting strategy involves shape memory polymers and bioactive components like hyaluronic acid, with a novel RGD-peptide-modified Gellan Gum bio-ink encapsulating primary cortical neurons, to study the cortical folding [201]. The development of more sophisticated organ-on-chip models that incorporate immune cells, glial cells with neurons, and their interactions with tissue microenvironments (e.g., blood, CSF fluidics) requires more improvement to model neuroinflammation and neuroimmune interactions more accurately [202]. The lack of vasculature in existing brain models limits their ability to mimic the complex interactions between the brain and the circulatory system. Integrating vascularization within brain organoids could mitigate the issue of nutrient accessibility and support the development of more physiologically relevant models [144]. By integrating the CSF on microfluidic devices, the human blood–cerebrospinal fluid barrier (hBCSFB) could be modeled to study neuroinflammation-related diseases by simulating the neuropathological conditions [203]. Additionally, high-throughput organs-on-a-chip systems incorporating automatic analytic tools and complicated designs for co-culturing multiple organs need to be more advanced to improve throughput and reduce errors caused by experiment operations between batches. The development and application of novel electronic platforms for long-term electrophysiological recording from brain organoids and assembloids have enabled chronic, non-invasive electrical recording for 3D brain organoids [204,205].

**Figure 4:**
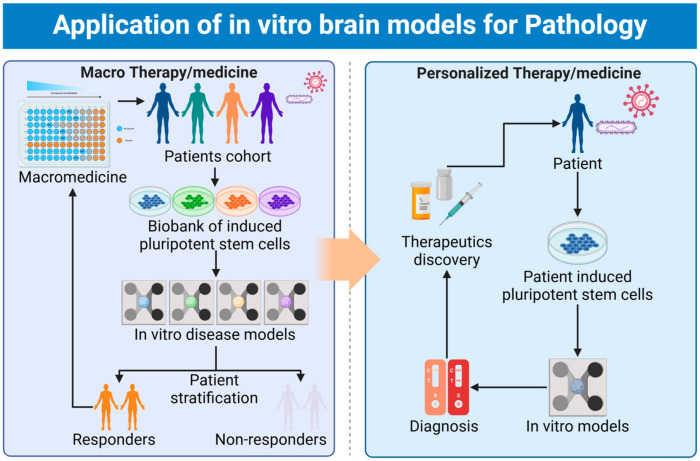
Application of in vitro brain models for therapeutics development. Traditional medicines are designed for the average patient without considering individual differences or through animal studies. In vitro human brain models will be utilized to stratify patient groups for better diagnosis, personalized medicine, and further application for precision medicine for broader patient prediction.

The in vitro human brain will be further utilized as a personalized and standarized platform for therapeutic discoveries in pathogen infection-driven neurodegeneration. (Figure 4).

### 5.1. Drug Development

Brain organoids and organs-on-chips systems provide more accurate and human-relevant information compared to traditional animal models. Organoids derived from patient tissues accurately recapitulate the complex microenvironment, genetic heterogeneity, and functional characteristics of the original organs, making them invaluable for personalized drug development and screening [206,207]. The integration of biosensors within these organoid systems facilitates the continuous monitoring of physiological responses to drugs, providing invaluable data on efficacy and safety in a more organ-specific context [208]. Organoids derived from various tissues have been co-cultured with a range of pathogens, including viruses, fungi, bacteria, and parasites, to mimic infection scenarios accurately, acting as a drug screening platform to identify potential anti-pathogen drugs and antibodies [202]. Organ-on-chips, on the other hand, enable high-throughput, dynamic, and combinatorial drug screening by facilitating the culture and analysis under precisely controlled conditions [209]. These systems are designed to automate the delivery of drugs in sequential or combinatorial formats, allowing for the exploration of the temporal effects of drug treatments on organoids derived from humans [210].

### 5.2. Gene Therapy

The CRISPR/Cas9 system has been a primary tool in enabling targeted genetic modifications within organoids, including the introduction of mutations or the correction of pathogenic variants, thereby providing a direct application in genetic therapy [211,212]. Organoids serve as an ideal platform for evaluating antisense oligonucleotide (ASO) therapies, enabling the study of disease-causing mutations within their native genetic context, and the application of the CRISPR-Cas9 system facilitates the generation of isogenic iPSCs, which can be used to introduce or correct disease-specific mutations, thereby providing a robust model for ASO assessment [213]. Brain organoids facilitate the introduction of multiple modifications at different locations simultaneously in the same cell, which is crucial for modeling the effects of multiple common variants in complex polygenic diseases [214].

### 5.3. Vaccine

Three-dimensional in vitro models have been utilized to replicate the complex architecture of lymph nodes and peripheral lymphoid organs, employing biocompatible materials and microfluidic devices to mimic in vivo fluid dynamics and mechanical stresses, thus enhancing the simulation of immune responses [215,216]. By integrating with a bioreactor system, the 3D in vitro models offer more physiologically relevant environments for vaccine testing, allowing for the co-culture of human B and T cells, forming micro-organoids upon stimulation by drugs or antigens, and can measure cytokine and antibody production [217]. Organoid culture, combined with next-generation sequencing (NGS) and single-cell RNA sequencing (scRNA-seq), facilitates the identification of therapeutic targets and mutation-associated neoantigens (MANAs) for novel targeted therapies and personalized vaccinations [218]. In vitro models are also crucial for evaluating the efficacy of adjuvant combinations in vaccine development, allowing for the assessment of antigen cross-presentation and T-cell activation [219].
